# Altered muscular activation during prone hip extension in women with and without low back pain

**DOI:** 10.1186/2045-709X-19-18

**Published:** 2011-08-14

**Authors:** Amir M Arab, Leila Ghamkhar, Mahnaz Emami, Mohammad R Nourbakhsh

**Affiliations:** 1 Department of Physical Therapy, University of Social Welfare and Rehabilitation Sciences, Evin, Tehran, Iran; 2 Student Research Committee, University of Social Welfare and Rehabilitation Sciences, Evin, Tehran, Iran; 3 Department of Physical Therapy, North Georgia College and State University, Dahlonega, GA, USA

**Keywords:** Electromyography, Low back pain, Movement pattern, Prone hip extension

## Abstract

**Background:**

Altered movement pattern has been associated with the development of low back pain (LBP). The purpose of this study was to investigate the activity pattern of the ipsilateral erector spinae (IES) and contralateral erectorspinae (CES), gluteus maximus (GM) and hamstring (HAM) muscles during prone hip extension (PHE) test in women with and without LBP. A cross-sectional non-experimental design was used.

**Methods:**

Convenience sample of 20 female participated in the study. Subjects were categorized into two groups: with LBP (n = 10) and without LBP (n = 10). The electromyography (EMG) signal amplitude of the tested muscles during PHE (normalized to maximum voluntary electrical activity (MVE)) was measured in the dominant lower extremity in all subjects.

**Results:**

Statistical analysis revealed greater normalized EMG signal amplitude in women with LBP compared to non-LBP women. There was significant difference in EMG activity of the IES (P = 0.03) and CES (P = 0.03) between two groups. However, no significant difference was found in EMG signals of the GM (P = 0.11) and HAM (P = 0.14) among two groups.

**Conclusion:**

The findings of this study demonstrated altered activation pattern of the lumbo-pelvic muscles during PHE in the women with chronic LBP. This information is important for investigators using PHE as either an evaluation tool or a rehabilitation exercise.

## Background

Low back pain (LBP) is one of the most common and costly musculoskeletal complaints in today's societies, affecting up to 70-80% of the population at least one episode during their lifetime [1,2]. Despite its high incidence and detrimental effects on individuals' activities, the exact causes of mechanical LBP have not yet been fully understood as any approach to diagnosis or treatment has been shown to be clearly effective. However, during the recent decades the approach in assessment and treatment of LBP has been progressed from strengthening of lumbo-pelvic muscles toward modification of the motor system [3]. Balanced motor system is resulted from coordinated activity of synergist and antagonist muscles. According to this point of view, repetitive movements and long-term faulty postures will change muscle tissue characteristics and can lead to muscle dysfunction, altered movement pattern, pain and finally movement disorders [3]. Increased or decreased muscle activity and delayed muscular activation can change the normal movement pattern [4,5]. Hence, the main focus has been recently placed on modification of the altered movement pattern in patients with musculoskeletal pain [4,6,7].

Several studies have demonstrated altered activation pattern of the certain lumbo-pelvic muscles during various tasks in people who suffer from LBP [8-11]. There are few clinical tests that assess the altered movement pattern in subjects with LBP. Prone hip extension (PHE) which has been developed by Janda is a common and widely accepted test for measuring the muscular activation pattern in the lumbo-pelvic area [4]. The importance of PHE is that the muscle activity pattern during this movement has been theorized to simulate those used during functional movement patterns such as gait [5,6]. It is thought that changes in this pattern can decrease the stability of lumbo-pelvic region during walking [12]. Good reliability has been reported for PHE in detecting deviation of lumbar spine from the midline [13].

The timing (onset time) and amplitude of muscle activation are commonly measured to assess muscular activation patterns in musculoskeletal disorders using electromyography (EMG) [14-17]. However, most previous studies have examined the timing of muscle activity during PHE in patients with LBP to determine the order in which the muscles are activated during this motor pattern [14-17].

To our knowledge, no study has investigated this motor pattern in order to determine the amplitude of lumbo-pelvic muscles activity in patients with chronic LBP. The purpose of this study was to investigate the amplitude of the activation pattern of the ipsilateral erectors pinae (IES), contralateral erector spinae (CES), ipsilateral gluteus maximus (GM) and ipsilateral hamstring (HAM) muscles during PHE in women with and without LBP and to compare time broadness among peak muscles activities in percent of total time of a movement cycle between groups.

## Methods

### Subjects

A cross sectional study design was used to compare the muscle activity pattern during PHE in two groups of women: women with chronic non-specific LBP (N = 10, average age: 33.6 (SD = 7.27) years old, average height: 163.1 (SD = 8.25) cm, average weight: 59.5 (SD = 10.34) kg) and women with no history of LBP (N = 10, average age: 29.8 (SD = 5.67) years old, average height: 161.2 (SD = 7.36) cm, average weight: 58.4 (SD = 5.44) kg). The LBP patients were referred by orthopedic specialist and physiotherapy clinics. The patients included if they have a history of non-specific LBP for more than 6 weeks duration before the study date, or intermittent LBP with at least three previous episodes lasting more than one week during the year before the study [18]. The healthy subjects were recruited from university students. The exclusion criteria in both groups were pregnancy, history of dyspnea, history of hip pain, dislocation or fracture, history of lumbar spine surgeries, history of anterior knee ligament injury or rupture, history of anterior knee pain, recent episodes of ankle sprain, leg length difference of more than 1 cm, inability to perform active PHE without pain, history of lower extremity injury in the past 3 months, shortness of hip flexors, those who participate in programs to prepare for competitive sports (exercise more than 3 days a week), positive neurological symptoms and cardiopulmonary disorders. Each eligible subject was enrolled after signing an informed consent form approved by the human subjects committee at the University of Social Welfare and Rehabilitation Sciences. Ethical approval for this study was granted from the internal ethics committee at the University of Social Welfare and Rehabilitation Sciences.

The dominant leg was chosen for investigation. The muscle activity of IES, CES, GM and HAM during PHE was measured by the MIE-MT8 Telemetry EMG instrument (MIE-Medical Research Ltd). A preamplifier with a gain of (4000×), band pass filtered (6-500 HZ), A-D converted (sampling rate = 1000 HZ) was used. The subjects were asked to lie prone with their arms at their side and head was in mid line. The skin was shaved, rubbed and cleaned with alcohol. To record muscle activity, disposable, bipolar, self adhesive Ag/Agcl electrodes were placed in pairs with distance of 1.5-2 cm from each other and parallel to the muscle fibers [19]. Electrodes placement to collect EMG signals were as follow: for the ES muscles, bilaterally at least 2 cm lateral to spinous process of L3 parallel to the vertebral column on the muscle belly; for the GM, at the mid point of a line running from S2 to the greater trochanter; and for the HAM, laterally on the mid distance between gluteal and popliteal fold.

The maximum voluntary electrical activity (MVE) for each muscle was firstly calculated for normalization procedure. Test methods to calculate MVE were similar to those described for manual muscle testing of the muscles, as described by Kendall et al [20]. The pelvis was secured to the bed with a sling to prevent pelvic motion substitution only during MVE testing. For the ES muscles the subject was asked to bring up her trunk against the maximum resistance that entered bellow the scapula. For the GM, hip joint was placed in extension position and knee flexed to 90 degrees, resistance applied to the distal aspect of posterior portion of thigh. The HAM was tested while hip joint was placing in extension position, the knee was flexed to nearly 70 degrees, and resistance was applied to the distal aspect of the posterior portion of the shank during knee flexion. Each contraction was repeated 2 times and held 5 seconds. One minute rest was given between contractions. Before testing, the subjects were familiarized with the standard position and movement. All subjects were asked to lift the chosen leg off the bed to 10 degrees whilst keeping the knee straight, as soon as they heard the command "lift". An adjustable bar was placed at this level and the subjects were asked to extend their hip until the calcaneous touched the bar. The subjects were instructed only to reach the adjustable bar and were not instructed to press against the bar with the distal segment of the lower extremity.

**Figure 1:**
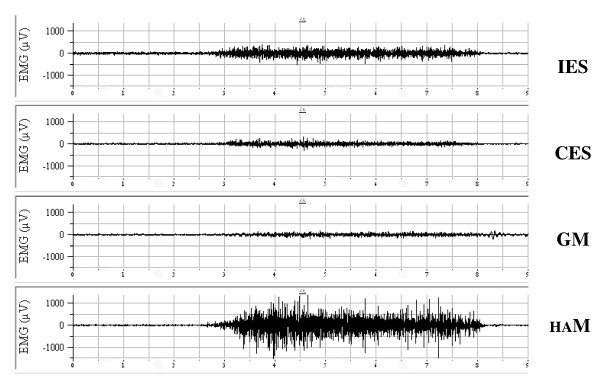
**Example of data recording from the tested muscles**.

This was repeated 3 times for each individual. Figure 1 depicts an example of the raw EMG signals for tested muscles. The raw data were processed into the root mean square (RMS). The EMG signals collected during hip extension were expressed as percentage of the calculated mean RMS of MVE (%MVE).

Time broadness is the time elapsed (in %) of the motion cycle between the peak of the first muscle to reach maximal activity and the peak of the last muscle to reach maximal activity. Time broadness can show to what extent the muscles are simultaneously involved in producing a motion during a motion cycle. Time broadness provides indirect information on muscle coordination [21]. The muscle activity pattern was characterized by maximal amplitude of normalized voluntary electrical activity and by time broadness in the percent of the movement cycle. The pattern is different in case there is a difference in any of the parameters above.

### Data Analysis

Statistical analysis was performed using SPSS version 15.0. Independent t-test was used to compare the maximal amplitude of normalized voluntary electrical activity of the tested muscles between women with and without LBP. Statistical significant was attributed to P value less than 0.05.

## Results

The demographic data for two individual groups are displayed in Table 1.

**Table 1:** Demographic data of the women in each group

Variables	With no LBP (n = 10)		With LBP (n = 10)		P-value
		
	Average (SD)	Median	Average (SD)	Median	
Age (years)	29.8 (5.67)	27.5	33.6 (7.27)	35	0.20
Weight (kg)	58.4 (5.44)	58.5	59.5 (10.34)	60.5	0.76
Height (cm)	161.2 (7.36)	160.5	163.1 (8.25)	161.5	0.59
BMI (kg/m^2^)	22.58 (2.88)	22.25	22.31 (3.31)	22.36	0.84

SD = Standard Deviation

LBP = Low Back Pain

BMI = Body Mass Index

There was no statistically significant difference in subjects' age, height, weight and BMI among the two groups.

The maximal amplitude of normalized electrical activity of the IES, CES, GM and HAM muscles during PHE test in women with and without LBP is presented in Table 2. There was significant difference in EMG activity of the IES (P = 0.03) and CES (P = 0.03) between two groups. The results indicated that normalized electrical activity of the muscles during PHE is higher in women with LBP compared to those without LBP. However, no significant difference was found EMG signals of the GM (P = 0.11) and HAM (P = 0.14) among two groups.

**Table 2:** Electromyographic activity of the muscles during prone hip extension in subjects with and without LBP

Muscle activity (%MVE)	With no LBP	With LBP	P-value
Ipsilateral Erector Spinea	46.86 (25.57)	70.74 (21.80)	0.03
Contralateral Erector Spinea	50.36 (20.25)	72.11 (24.10)	0.04
Gluteus Maximus	29.81 (14.14)	42.32 (18.93)	0.11
Hamstring	52.78 (33.44)	74.06 (28.69)	0.14

Values are Mean (SD)

LBP = Low Back Pain

## Discussion

The current study compared lumbo-pelvic muscle activation pattern between subjects with and without LBP. The results of this study showed higher maximal amplitude of normalized electrical activity of the IES, CES in patients with chronic LBP compared to those without LBP. The normalized electrical activity of the GM and HAM, although not statistically significant, was greater in women with LBP than healthy subjects. These findings demonstrate an altered activity pattern of the lumbo-pelvic muscles during hip extension in patients with chronic LBP. In this study, none of the subjects reported that pain was a limiting factor to perform PHE test, so, direct effects of pain can be minimized. However, nocioception can influence muscle activity. Bruno et al [15] studied the PHE movement pattern difference between subjects with and without LBP, measuring onset time of the EMG activity in IES, CES, GM and HAM. They found delayed activation of the GM during PHE in patients with unilateral LBP and concluded that the movement pattern is changed in LBP [15].

In many other studies, increased signal EMG amplitude of trunk muscle has been shown in patients with LBP during functional activities such as bending the trunk forward, back ward and gait [22-26]. In contrast, some studies showed vague results or even reduced signal EMG amplitudes [27]. Some of these differences can be explained by methodological problems, an important one of them is how the data is normalized. Many factors affect on absolute EMG amplitudes, such as thickness of tissues overlying the muscle and skin impedance. To obtain a net signal that is independent of these factors, the EMG amplitude must be normalized to the amplitudes obtained in MVE. However, this procedure may not be appropriate for patients because they usually unwilling or not able to perform maximum contractions due to pain or fear of re-creating pain. Normalization to sub maximal contractions is not a good way because the EMG amplitudes during these contractions will be affected similarly to the levels during the activities to be studied. In current study, MVE method was used because patients had no pain during the test.

It is commonly believed that lumbo-pelvic instability is an important component in chronic LBP. Investigators have attributed the increased activity of trunk muscles found in patients with LBP to functional adaptations following reduced spinal stability in these patients [26]. The spinal stabilizing system was primarily described by Panjabi [28], including of 3 subsystems: the spinal column providing intrinsic stability; spinal muscles, providing dynamic stability and neural control unit controlling and determining the requirements for stability and coordinating the muscle responses [28]. Under normal situations, the three sub systems work in harmony and provide the needed mechanical stability [29,30]. It seems that the spinal instability as a result of dysfunction of spinal structures or decreased neural control is compensated by increasing trunk muscle activity [28]. Co-contraction of ES muscles could be used to compensate the loss of passive stability [22,31,32]. Muscles can contribute to increase stability of trunk through co-contraction [31,33,34]. An alternative explanation might be that in the spine, the local stabilizers muscles (e.g. Tr.A) contract first then global stabilizer (e.g. ES), and acting as synergist to increase the stability in times of extreme need. With pain, injury or other pathologies an abnormal stabilizer recruitment pattern can be developed [35]. In this case, the activity of global stabilizer muscles will increase significantly to compensate the deep local muscles dysfunction and decreased spinal stability. Increased activity of ES, could cause pain in muscles themselves, contribute to vicious circle of pain-spasm-pain. In addition, co-contraction of trunk muscles would increase the loads on the spine [36].

Increased GM activity, although not statistically significant, was found in subjects with LBP. According to Van Wingerden [37], GM has an important role in sacroiliac joint (SIJ) stability because of its perpendicular fibers to the SIJ. Therefore, any pain and pelvic instability can lead to increased muscle activity especially in tasks that are required hip extension to enhance the SIJ stability. However, about 2-20% of the patients suffering from LBP have SIJ dysfunctions [38], while most of the patients in this study demonstrated increased GM muscle EMG activity. However, in this study we did not differentiate the SIJ pain. More research is needed to resolve the existing ambiguities in this area.

Increased activity of the HAM in women with LBP may be due to high fatigability [39] and poor endurance of the lumbar ES muscles [40,41]. As a result, increased HAM activity is an adaptive mechanism following lumbar muscles fatigue and possibly weakness in those muscles [42]. GM, BF, ES and latissimus dorsi are the key structures in providing SIJ stability [43]. Decrease in endurance of ES in subjects with LBP may relax the sacrotuberous ligament which is considered as the primary stabilizer structure in the SIJ [44]. The HAM can affect on sacrotuberous ligament by its proximal attachment to this ligament. It is thought that increased HAM activity in patients with LBP may be a compensatory functional mechanism resulting from this situation [44]. Considering difference in muscle activity pattern during PHE between subjects with and without LBP, PHE can be used as either an evaluation tool or a rehabilitation exercise for the subjects with LBP.

However, we acknowledge several important limitations. One of the limitations and weakness of this study was the sample size.

One point must be considered with regard to generalizing the present results, is the sample population. In this study, only women were recruited and men were not included. Therefore the results of this study may be more applicable to female subjects, who constituted the participants and could not be extrapolated to the men. It is suggested to perform this study in men to compare data between men and women.

EMG measurements do not always guarantee magnitude of force production and therefore muscle strength, as in some cases an inhibited muscle may be working harder than normal to produce the required force for a particular task. The timing of muscle activity in addition to EMG amplitude can provide more useful information regarding the muscular activation pattern.

Another area of concern in our study was this issue that LBP women were not categorized as with or without SIJ involvement.

## Conclusions

The results of this study indicate higher maximal amplitude of normalized electrical activity of the IES, CES in patients with chronic LBP compared to those without LBP. The normalized electrical activity of the GM and HAM, although not statistically significant, was also greater in women with LBP than healthy subjects. These findings demonstrate an altered activity pattern of the lumbo-pelvic muscles during hip extension in patients with chronic LBP. This information is important for investigators using PHE as either an evaluation tool or a rehabilitation exercise.

## List of abbreviations

LBP: Low Back Pain; IES: Ipsilateral erector spinae; CES: Contralateral erector spinae; GM: Gluteus maximus; HAM: Hamstring; PHE: Prone hip extension; EMG: Electromyography; MVE: Maximum voluntary electrical activity.

## Competing interests

The authors declare that they have no competing interests.

## Authors' contributions

AMA contributed to conception, design, analysis, interpretation of data and drafting the manuscript. LG carried out the data collection and involved in interpretation of data and drafting the manuscript. ME participated in data collection and analysis of EMG signals. MRN participated in design and helped to draft the manuscript. All authors read and approved the final manuscript.
